# Flow study of Dean’s instability in high aspect ratio microchannels

**DOI:** 10.1038/s41598-023-44969-9

**Published:** 2023-10-19

**Authors:** Yu Ching Wong, Cheng Dai, Qingyue Xian, Zhaoxu Yan, Ziyi Zhang, Weijia Wen

**Affiliations:** 1 Department of Physics, The Hong Kong University of Science and Technology, Clear Water Bay, Hong Kong; 2 Division of Emerging Interdisciplinary Areas, The Hong Kong University of Science and Technology, Clear Water Bay, Hong Kong; 3 Thrust of Advanced Materials, The Hong Kong University of Science and Technology (Guangzhou), Guangzhou, China; 4 HKUST Shenzhen-Hong Kong Collaborative Innovation Research Institute, Futian, Shenzhen, China

**Keywords:** Engineering, Materials science, Physics, Fluid dynamics

## Abstract

Dean’s flow and Dean’s instability have always been important concepts in the inertial microfluidics. Curved channels are widely used for applications like mixing and sorting but are limited to Dean’s flow only. This work first reports the Dean’s instability flow in high aspect ratio channels on the deka-microns level for $De>162$. A new channel geometry (the tortuous channel), which creates a rolled-up velocity profile, is presented and studied experimentally and numerically along with other three typical channel geometries at Dean’s flow condition and Dean’s instability condition. The tortuous channel generates a higher *De* environment at the same *Re* compared to the other channels and allows easier Dean’s instability creation. We further demonstrate the use of multiple vortexes for mixing. The mixing efficiency is considered among different channel patterns and the tortuous channel outperforms the others. This work offers more understanding of the creation of Dean’s instability at high aspect ratio channels and the effect of channel geometry on it. Ultimately, it demonstrates the potential for applications like mixing and cell sorting.

## Introduction

The field of inertial microfluidic has been gaining attention in recent years^1–3^. Inertial microfluidics is distinctly different from conventional microfluidics. Conventional microfluidics lies in the Stokes’ flow regime ($Re\rightarrow 0, Re=\frac{\rho UH}{\mu }$, where $\rho$, *U*, *H* and $\mu$ are the fluid density, average fluid velocity, channel dimension and dynamic viscosity) while inertial microfluidics works in an intermediate scale of *Re*, which ranges from ten to hundreds. The high throughput leads to non-negligible inertia in principle and short operation time in practice. This feature also ensures channel simplicity, allowing easy integration with other systems in applications like particle and cell sorting.

Dean’s flow (secondary flow), the generation of two counter-rotating vortexes in the plane perpendicular to the main flow in a curved channel, is an essential concept in inertial microfluidics. Dean’s flow microfluidics devices were used as a sorter^4–6^ or a mixer^7–9^.

Taylor^10^ first investigated secondary fluid instability with curved streamline mathematically. Dean^11^ and Reid^12^ later considered this problem in a curved cylindrical channel with $d\ll R$ (*d*: diameter, *R*: radius of curvature). Dean’s instability (secondary instability) occurs when Dean’s number (*De*) ($De=Re\sqrt{\frac{H}{2R}}$) exceeds a critical value, in addition to the influence by aspect ratio^13^. Multiple numerical studies were done to investigate Dean’s instability and the factors affecting the establishment of vortexes^14–17^. Experimental Dean’s instability studies were mainly carried out at the macroscale, where the visualisation of the cross-section is easier to achieve. Sugiyama et al.^18^ obtained secondary flow images in the curved 25 $\upmu$m wide rectangular channels in a range of aspect ratio (0.5–2.5) and curvature ratio (5–8) to find out the critical *De*. Bara et al.^19^ investigated the curved square duct with 1.27 square cross-section at different *De* (125, 137 and 150) and curvature ratio of 15.1 every 20° for detailed development of secondary flow.

Experimental work on Dean’s instability at the microscale relies on confocal microscopy. Nivedita et al.^20^ first visualized the secondary flow pattern of low aspect ratio channels in different aspect ratios with hecto-micron length for their critical *De* and reported the shift of particles towards the concave wall as Dean’s instability emerged.

**Figure 1:**
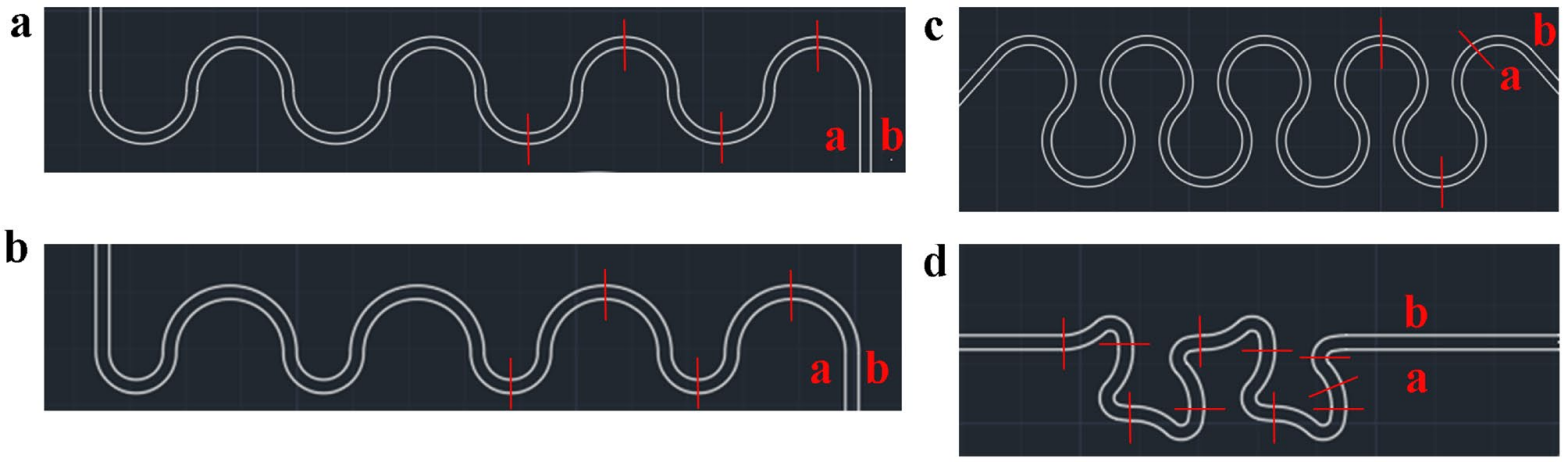
Channel patterns [Right-hand side (RHS): inlet, Left-hand side (LHS): outlet] (**a**) Symmetric channel. (**b**) Asymmetric channel. (**c**) Helical channel. (**d**) Tortuous channel. Sidewalls a and b are defined as shown above. Red lines are the observed positions (OPs).

In this paper, a study illustrating the generation of Dean’s instability in a high aspect ratio channel on deka-micron scale is presented. Three typical channel geometries^21,22^ and one new design with same width (60 $\upmu$m) and aspect ratio (1.5) are investigated (Fig. 1). Apart from study the fluid flow, the patterns are compared as a micro-mixer.

## Dean’s flow and Dean’s instability

Dean’s flow develops in a curved channel at an intermediate range of *Re*. The usual cross-sectional velocity profile in such a device is paraboloid in nature. Considering the fluid unit elements at the centre of the cross-section as they move in the curved channel, they experience a centrifugal force:

1 $$\begin{aligned} F_c=\frac{d\rho U^2}{R} \end{aligned}$$

Due to the velocity profile nature, fluid elements at the centre of the cross-section have the highest velocity and experience the greatest centrifugal force. The fastest fluid elements are pushed towards the outer wall and recirculate. Hence, two counter-rotating Dean’s vortexes generate.

As the flow rate increases and attains the critical *De*. The radial pressure gradient becomes non-negligible and dominates over the centrifugal force near the outer wall region. The radial pressure gradient is:

2 $$\begin{aligned} P_r=\frac{\Delta p}{H} \end{aligned}$$

where $\Delta p$ is the pressure difference along the radial axis between the sidewalls. New vortexes generate in the pressure gradient-dominated region and fluid elements travel from the outer wall towards the inner wall. Near the boundary of balance between pressure gradient and centrifugal force, fluid elements are pushed back and then recirculate. As a result, a pair of centrifugal force-generated vortexes and another pair of pressure gradient-generated vortexes co-exist.

To relate the centrifugal force and the radial pressure gradient, a dimensionless parameter $\Omega$ is derived by taking a ratio. $\Omega$ offers a way to compare the strength and size of vortexes when Dean’s instability occurs. The equation of $\Omega$ is:

3 $$\begin{aligned} \Omega = \frac{\Delta p/H}{\rho U^2/R} \end{aligned}$$

Another way to derive the $\Omega$ is by the Buckingham-Pi theorem^23^.

## Results and discussion

### Symmetric channel

**Figure 2:**
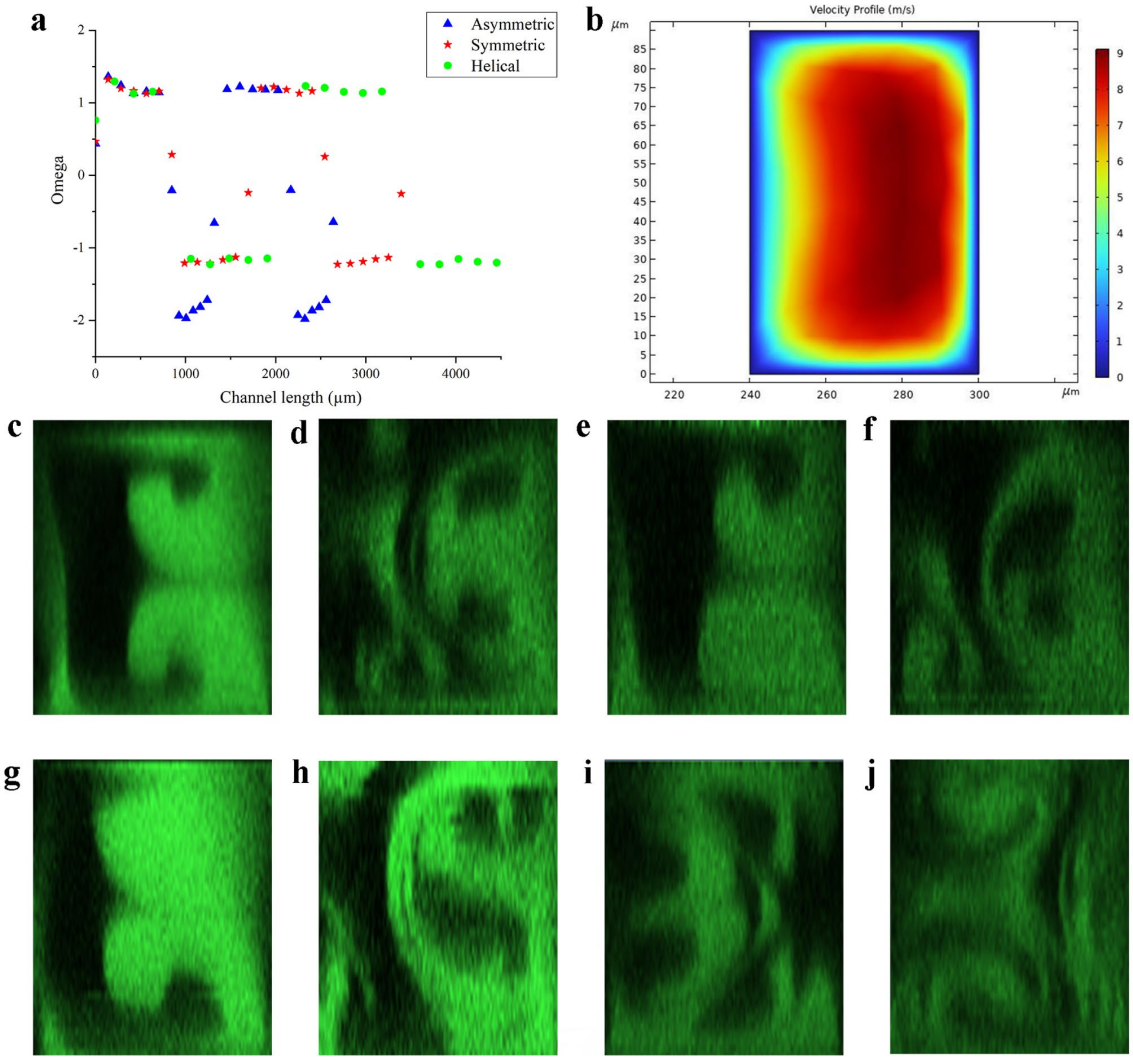
(**a**) Omega $\Omega$ versus channel length in the first four turns at $Re=444$ for symmetric, asymmetric and helical channels. (**b**) Velocity profile of the cross-section at 2nd OP of symmetric channel. Cross-sectional image of the channels at $Re=444$, $De=162$ (LHS: sidewall b, RHS: sidewall a): (**c**) Symmetric: 1st OP. (**d**) Symmetric: 2nd OP. (**e**) Asymmetric: 1st OP. (**f**) Asymmetric: 2nd OP at $De=218$. (**g**) Helical: 1st OP. (**h**) Helical: 2nd OP. (**i**) Helical (changing the first turn to 270°): 1st OP. (**j**) Helical (changing the first turn to 270°): 2nd OP.

As shown in Fig. 2a, the average $\Omega$ at the first and second turn are 1.19 and − 1.18. The change in the sign after 180°indicates the change in the direction of the radial pressure gradient. We observe two pairs of anti-rotating vortexes in the second turn as shown in Fig. 2d. The pair next to sidewall a exists due to the imbalance of radial pressure gradient to centrifugal force. Another pair of vortexes near sidewall b arises from the effect of centrifugal force. This pair of vortexes is forced towards the horizontal walls.

**Figure 3:**
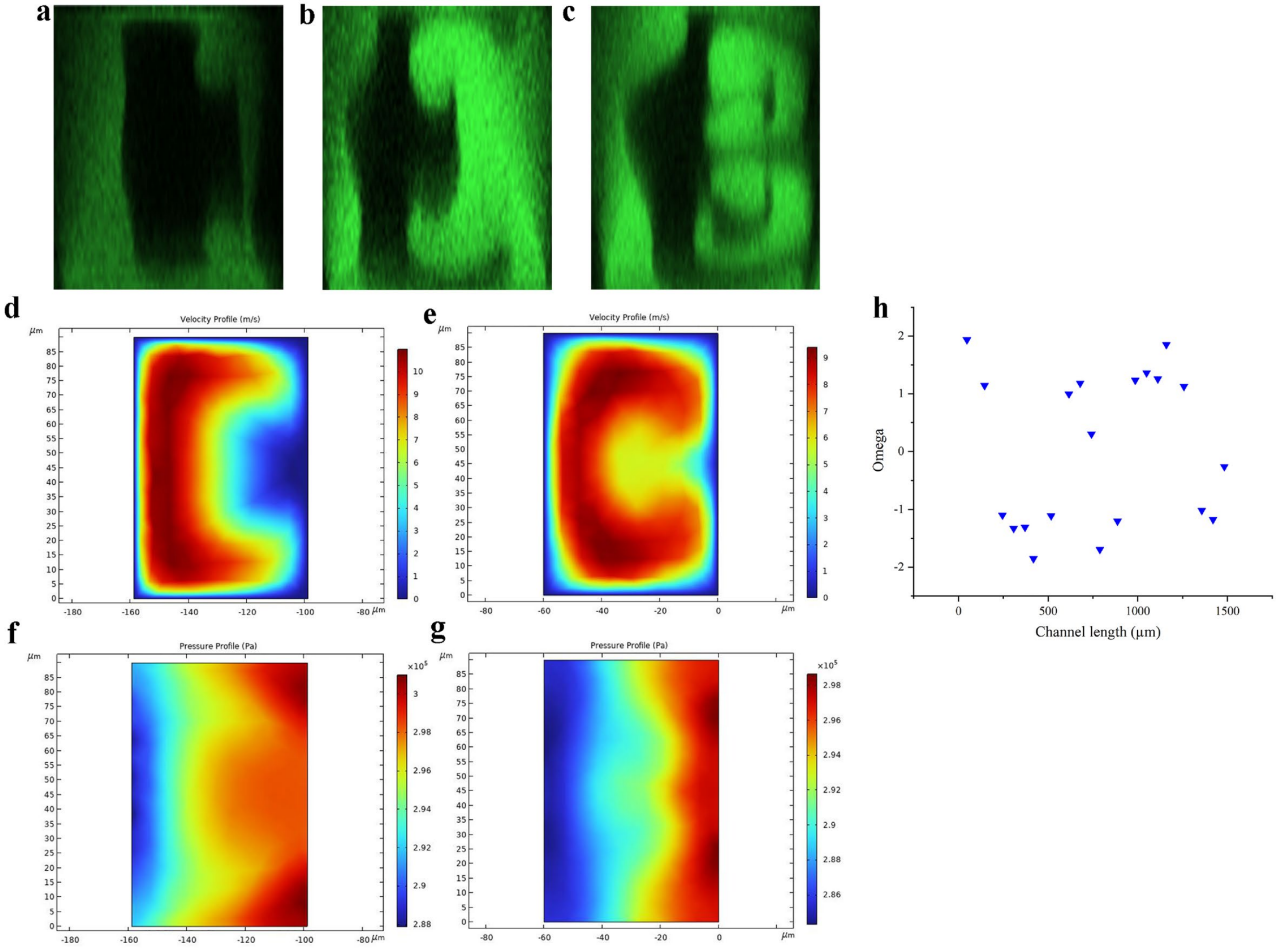
Cross-sectional image of the tortuous channels at $Re=444$ (LHS: sidewall b, RHS: sidewall a): (**a**) 1st OP at $De=172$. (**b**) 2nd OP at $De=344$. (**c**) 3rd OP at $De=162$. (**d**) Velocity profile of the cross-section at 2nd OP. (**e**) Velocity profile of the cross-section at 3rd OP. (**f**) Pressure profile of the cross-section at 2nd OP. (**g**) Pressure profile of the cross-section at 3rd OP. (**h**) Omega $\Omega$ versus channel length in the first four units at $Re=444$ in the tortuous channel.

The distorted velocity profile of the second turn in Fig. 2b explains such a phenomenon. The fluid elements with higher velocity are pushed from the channel centre towards sidewall a. The fluid elements near the corners of sidewall b are faster than that near the middle of sidewall b. The radial pressure gradient dominates over centrifugal force near the sidewall a region and the fluid elements near the sidewall b corner are responsible for generating vortexes by centrifugal force.

A pair of anti-rotating vortexes is observed in the first turn in Fig. 2c. This pair of vortexes evolves from the two corners at sidewall a, and it matches the direction of the vortexes generated by centrifugal force. An incomplete vortex pattern is observed because the position of the first confocal image taken is at 90 degrees. That means the flow is undeveloped and is consistent with previous observations, which stated it took 240 degrees to attain a fully developed state at $De=150$^19^.

### Asymmetric channel

Figure 2e shows the cross-sectional image in the first turn. It is similar to the symmetric channel as they have the same geometry. Figure 2f presents the confocal image in the next turn. A partially similar pattern to that in the symmetric channel is observed. The differences are the sizes of the vortexes and the loss of a vortex at the top corner close to sidewall b. The ratio of the size of vortexes near the sidewall a to the sidewall b in the asymmetric channel (1.632) is larger than that in the symmetric channel (1.399). It is due to the higher radial pressure difference at a greater curvature, resulting in a greater $\Omega$ at the second turn. As shown in Fig. 2a, the average $\Omega$ at the first turn is 1.20, and that at R = 150 $\upmu$m is − 1.86. As the pressure gradient reverses in the radial direction, the sign of $\Omega$ flips for every 180°.

The loss of the top corner vortex at sidewall b is unexpected. We think the difference between top and bottom wall material is accountable for that. The bottom surface is hydrophilic glass, while the top surface is hydrophobic PDMS. Hence, the asymmetric wettability leads to the loss of the top corner vortex.

### Helical channel

The secondary flow pattern of the helical channel is similar to that of the symmetric and asymmetric channels in the first two turns (Fig. 2g,h). The ratio of the size of vortexes near the sidewall a to the sidewall b in the helical channel is 1.44. This value is close to that of the symmetric channel but smaller than that of the asymmetric one. That is reasonable because the helical channel shares the same radius as the symmetric channel. The average $\Omega$ at the first turn is 1.19, and that at the second turn is − 1.17 as shown in Fig. 2a, the sign of $\Omega$ flips after 180°in the first turn and every 270°afterwards.

The bending angle affects vorticity as we mentioned. Such a phenomenon can not be observed in the secondary flow patterns presented because the channel’s first turns are identical for the three patterns discussed above. Instead of looking into the cross-section image in the third turn of the helical channel because the flow there will be too chaotic, we fabricated a helical channel with the first turn changed to 270°. Two pairs of vortexes can be observed in the first turn (Fig. 2i). The original pattern is single pair of vortexes due to undeveloped flow, with the elongation of bending and the change in OP from 90°after the main channel inlet to 135°after the main channel inlet. Now the flow is more developed, and it explains why two pairs of vortexes is seen.

There is only one pair of vortexes which is generated by centrifugal force in the second turn from Fig. 2j. The effect of the radial pressure gradient is hard to notice, but it does exist as the fluid in the middle of sidewall a is slightly pushed towards sidewall b. We think the stronger swirl entering the second turn reinforces the centrifugal force-generated vortexes. And the unchanged radius makes the pressure gradient less dominant near the outer wall region. That leads to the generation of single pairs of vortexes.

**Figure 4:**
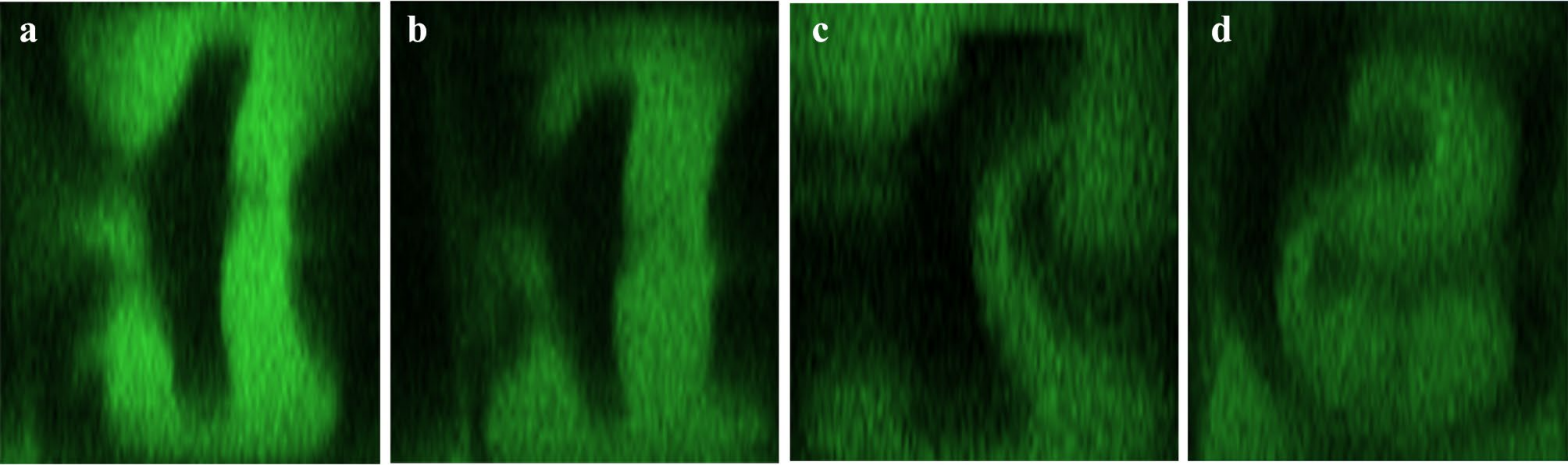
Cross-sectional images at $Re=222$ at 2nd OP for all channels (LHS: sidewall b, RHS: sidewall a): (**a**) Symmetric channel. (**b**) Asymmetric channel. (**c**) Helical channel. (**d**) Tortuous channel.

**Figure 5:**
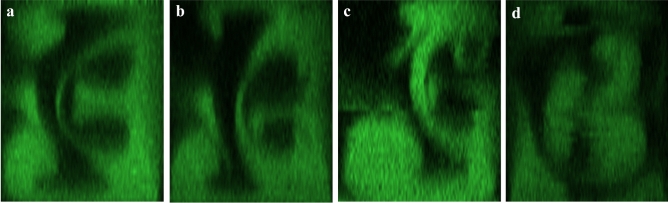
Cross-sectional images at $Re=333$ at 2nd OP for all channels (LHS: sidewall b, RHS: sidewall a): (**a**) Symmetric channel. (**b**) Asymmetric channel. (**c**) Helical channel. (**d**) Tortuous channel.

### Tortuous channel

The first confocal image is taken in the middle of the second turn in the first unit. The secondary flow starts to develop at this point. In Fig. 3a, the fluorescent elements are suppressed towards sidewall b and evolve from the corners near sidewall a under the centrifugal effect.

As the fluid leaves the second turn, the velocity profile (Fig. 3d) is distorted similarly to the symmetric channels but to a greater extent as the fluid elements with higher velocity move to and elongate along the sidewall. Along sidewall b, the centrifugal forces are comparable, and the pressure gradient is stronger along the top and bottom sides as shown in Fig. 3f. The stronger pressure gradient leads to a smaller net force along the top and bottom, resulting in the net movement of fluid from sidewall b to A along the horizontal mid-line. Thus, a pair of counter-rotating vortexes is formed (Fig. 3b).

The velocity profile is further distorted and rolled up shown in Fig. 3e as the fluid proceeds in the third turn. The fluid elements with higher velocity translate to the top and bottom sides, indicating that the centrifugal forces there are stronger. Moreover, due to the rolled-up velocity profile, the pressure gradient along the middle of the cross-section is reinforced (Fig. 3g), which presents a larger contrast between radial pressure gradient and centrifugal force. As a result, two new vortexes arise along the middle while the top and bottom vortexes remain due to carried momentum as shown in Fig. 3c. The $\Omega$ value in this design attains up to 1.94 (Fig. 3h), meaning a greater imbalance between centrifugal force and radial pressure gradient can be achieved.

The difference in secondary flow behaviour in tortuous channels is mainly due to the rolled-up velocity profile. Instead of having two distinct pairs of vortexes generated by centrifugal force and pressure gradient respectively, now in the tortuous channel, the centrifugal force and pressure gradient “cooperate” for vortexes formation. We believe such a phenomenon has a meaningful impact on particle separation and mixing.

### The dependency of flow rate and wettability on flow behaviour

The above sections discussed the flow at Dean’s instability regime ($Re=444$) in different patterns, this section analyzes the effect of flow rate and wettability. Fig. 4a–c are the cross-sectional images of symmetric and asymmetric channels, and the helical channel at the second turns at $Re=222$. Only two vortex remains in the second turn. The vortex in the helical channel is slightly distorted, but no complete vortex by the pressure gradient is observed. The De is now near the critical De due to the drop in flow rate, and instability is vanishing/weakening. Hence, we concluded Dean’s flow presented in symmetric, asymmetric and helical channels at $Re=222$.

Figure 5a–c are the cross-sectional images of symmetric, asymmetric channels, and the helical channel at the second turns at $Re=333$. From these three graphs, we observe that Dean’s instability emerges and the vortexes start to generate and get closer to the flow we saw at $Re=444$. However, the vortex is not visible enough as compared to that at $Re=444$ as there is only a slight curling up of the fluid. We also observe the wettability affects the flow significantly as there is a loss of top vortex in Fig. 5b,c similar to Fig. 2f. At $Re=333$, we believe flow is transiting from Dean’s flow to Dean’s instability as pressure-gradient generated vortexes are now observable for symmetric, asymmetric and helical channels.

While for the flow in the tortuous channel, as shown in Fig. 4d, four vortexes still exist at the end of the first unit at 1000 $\upmu$l/min. Although the number of vortexes is unchanged, an asymmetric pattern is observed. This is interesting as we observe that along the horizontal mid-line, the lower fluid element is pushed from RHS to LHS first and followed by the upper fluid element, making the earlier creation of the bottom vortex. We believe this is the difference in wettability we mentioned above. As the *Re* increases to $Re=333$ as shown in Fig. 5d, asymmetricity disappears. Overall, we concluded Dean’s instability emerges in the tortuous channel at both $Re=222$ and $Re=333$.

The reason that tortuous design could sustain instability in a lower flow rate is because of the perturbation of the second turn, which has a radius of 60 $\upmu$m. That means the centrifugal force in the second turn is at least 16 times more than that in the other turns. Hence, the tortuous channel allows the creation of secondary instability at a lower flow rate compared to the other channels.

From all the patterns we presented and analyzed, we observed that wettability affects the flow to three extents, one is the loss of vortex as we observe in Figs. 5b,c and 2f. Another one is the rise of obvious asymmetric flow patterns as we see in Fig. 4d. And the last one is the slight asymmetricity which we did not mention above as shown in Figs. 2d,e,g,h and 3c. And there is an overall trend that the effect of wettability is weakening as the flow rate increases and there finally exists only slight asymmetricity. We believe the asymmetricity may eventually become unnoticeable, for example, Figs. 2c and 4a, as the flow rate increases even more. However, such a phenomenon is inevitable due to the nature of the material. To fully avoid the effect of wettability, the fabrication process has to be modified but the existing solutions either post difficulties in fabrication or challenges in data collection.

**Figure 6:**
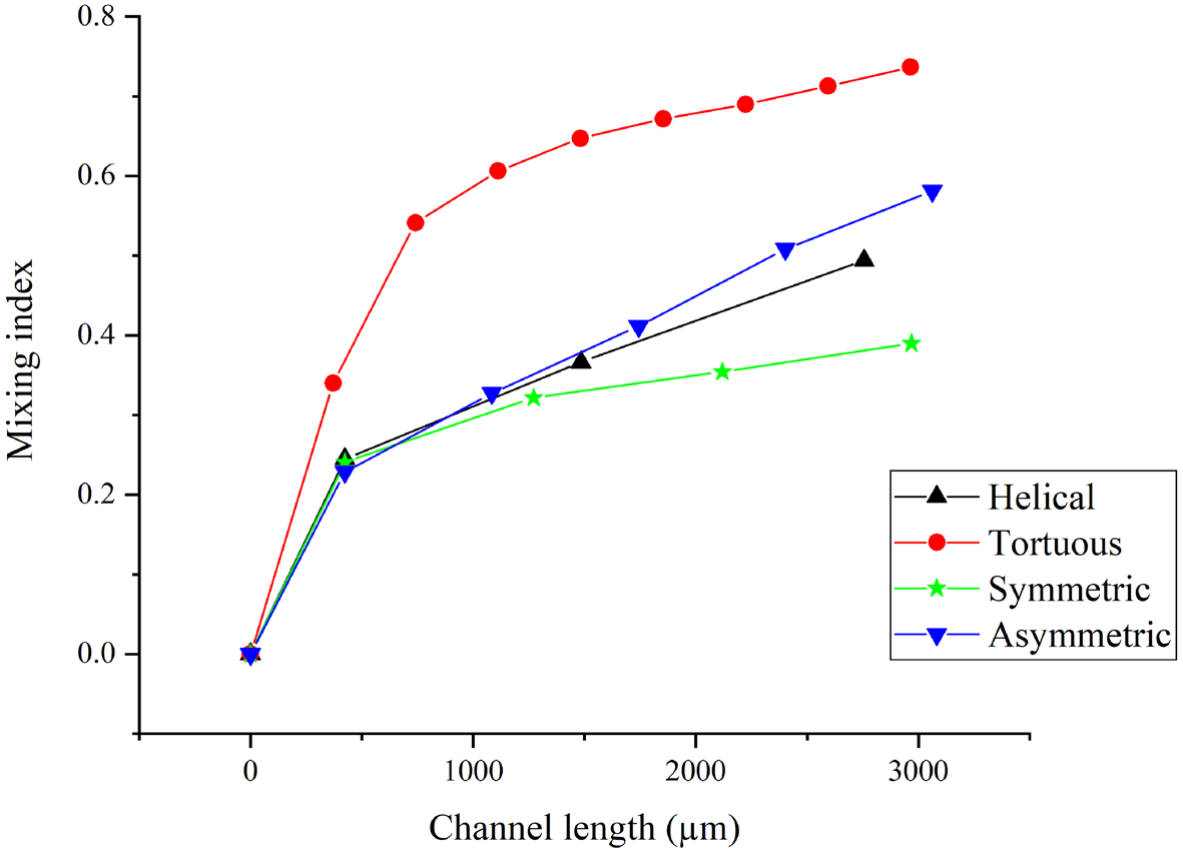
Mixing indices versus channel length in all patterns.

### Comparison as a micro-mixer at $Re=444$

The MI is plotted against the channel length, and we are comparing the mixing efficiency across all the patterns by MI per unit length. As shown in Fig. 6, the slopes of the symmetric, asymmetric and helical channels at the start of the curve are close ($\sim$6E−5) due to similar geometry at the beginning of the channel. The curves deviate in the later part, and the corresponding slopes are 4E−5 (symmetric channel), 11E−5 (helical channel) and 13E−5 (asymmetric channel). Although the condition for the Dean’s instability and the flow behaviour are similar in these three patterns, the MI suggests that the effect of the radius of curvature (centrifugal force) is more important than the bending angle (swirl in the next turn) in terms of mixing. The MI per unit length further verifies the above statement: symmetric channel (1.31E−4), asymmetric channel (1.90E−4), and helical channel (1.88E−4).

The curve of MI in the tortuous channel has a sharp rise with slope (91.7E−5) and is followed by a steady increase with slope (8.11E−5). The MI per unit length attains 2.48E−4 which outperforms the other patterns. The tortuous channel is superior in mixing as it combines the benefits of stronger centrifugal force and swirl with the perturbation of the second turn in each unit. It also enhances mixing by providing more reversal of direction in centrifugal force and pressure gradient throughout the channel.

## Methods

### Channel design

Four patterns are used: symmetric channel, asymmetric channel, helical channel, and tortuous channel as shown in Fig. 1. The width of these channels is 60 $\upmu$m with an aspect ratio of 1.5. The symmetric channel (Fig. 1a) has a radius of 270 $\upmu$m, and the asymmetric channel (Fig. 1b) changes its radius to 150 $\upmu$m in even number turns. The helical channel (Fig. 1c) has the same radius as the symmetric channel but a different angle (270°) for every turn except the first and the last one. The effect of the angle of bending and the radius of curvature are compared with these three commonly used patterns.

The tortuous channel (Fig. 1d) is a pattern designed specifically for easier Dean’s instability generation. There are three radii of curvatures (270 $\upmu$m, 60 $\upmu$m and 240 $\upmu$m) in a single unit. And the second curvature acts as a perturbation that a greater centrifugal force is exerted on the fluid there. Another idea is that a larger angle can be achieved in a shorter channel length with a smaller radius to increase the axial vorticity according to Squire and Winter formula^24^, which states the axial vorticity is velocity gradient ($\partial u/\partial z$) times the angle of bending ($\epsilon$) with a coefficient of − 2.

### Fabrication

The devices were fabricated in two stages as shown in the supplementary document. In the first stage, the fabrication of the silicon mould for polydimethylsiloxane (PDMS) was carried out at The Hong Kong University of Science and Technology Nanosystem Fabrication Facility (HKUST NFF) using standard lithography and deep reactive ion etching (DRIE). 3 $\upmu$m HPR 506 was spun, exposed in Karl Suss MA6 and developed by the FHD5 developer. It was used as a mask for etching in the DRIE Etcher. The etching depth across the silicon wafer was 90 $\upmu$m ± 2$\upmu$m with multiple measurements by Tencor P-10 Surface Profiler, but the height of the single pattern was about the same. The second stage was done in a typical laboratory environment. 10:1 PDMS of 50 g was poured onto the mould and cured in the oven at 80 $^{\circ }\hbox {C}$ for 24 h. The glass slide (SLItech, microscope slides, MS-13) was bonded to the PDMS after 3 min of plasma treatment in the plasma cleaner (Harrick, PDC-001-HP) followed by baking at 80 $^{\circ }\hbox {C}$. After inlets and outlets drilling and tubing (Tygon Microbore Autoanalysis Tubing, GM-06419-03) connection, the junction sealing was achieved by optical adhesion (Norland optical adhesive 61) with 2 h of UV light exposure in the light curing system (DYMAX, Model 2000 Flood) followed by 8 h of baking at 80 $^{\circ }\hbox {C}$.

### Sample preparation

8-Hydroxypyrene-1,3,6-trisulfonic acid trisodium salt (HPTS) purchased from Sigma-Aldrich was used as the fluorescence. 2.75mM HPTS solution was prepared by mixing 0.05g of HPTS with 35 ml of deionized (DI) water. The HPTS solution was used to perform mixing with DI water.

### Experimental setup and data analysis

The experiment setup is shown in the supplementary document. The infusion was done by two pumps (Harvard Apparatus, PHD 2000 Infusion) and three different total flow rates: 1000 $\upmu$L/min ($Re=222$), 1500 $\upmu$L/min ($Re=333$) and 2000 $\upmu$L/min ($Re=444$) were studied. Initial simulation was carried out to compare the streamline among all channels and it was found that only Dean’s instability occurred in the tortuous channel at a flow rate of 1000 $\upmu$L/min ($Re=222$). Hence, 1000 $\upmu$L/min ($Re=222$) was chosen as the starting point. The 2000 $\upmu$L/min ($Re=444$) was close to the upper flow rate limit we achieved experimentally. We found that leakage happened in the helical channel every time the flow rate approached 2300 $\upmu$L/min. We believed that was because of the relatively higher resistance in the helical channel compared to the other channels due to a longer channel length.

The observation was achieved by xyz scanning of the confocal microscope (Leica TCS SP5). The observed channel positions are the middle of turns for the symmetric, asymmetric and helical channels. For the tortuous channel, except the first channel unit was recorded in detail, the near-ends were observed for the remaining units. The laser source was a gas-state Argon laser with multiple wavelengths. The confocal microscopy parameters are attached in the supplementary document. 458 nm was selected for HPTS observation. Steps size of 1.83 $\upmu$m was used for consistency throughout the scanning of different samples. The step size resolution depends on the pinhole, it increases the optical section thickness and received signals from the adjacent layers. Gains simply amplify the signal received by the image sensor. Although the environment light and concentration of fluid were controlled and maintained the same throughout the experiment, the power of the gas-state laser was less stable than a solid-state laser and affected the signal. Gain and pinhole were varied to give the best image quality. The offset affects the image saturation and − 0.2% was suggested by the Leica company’s engineer. Line and frame averages were used for decreasing the background noise and increasing contrast while the accumulation was used for a stronger signal. Different averages and accumulations were observed and found out the value in the table was sufficient. It is better for a higher value, but the time cost increases as well. Overall, each scan across the z-axis lasted for about 30 min with a scanning frequency of 400 Hz. The obtained image was then processed by ImageJ, and graphs were plotted by OriginLab. The sizes of vortexes were also analysed on ImageJ by measuring the radius of the vortex from the vortex core.

### Mixing index (MI)

Usually, the standard deviation calculates the MI. However, the standard deviation cannot accurately compare different images as there are differences in confocal settings^25^. The coefficient of variation is used to calculate the MI for a fairer comparison between the devices. The equation of the MI is:

4 $$\begin{aligned} RMI=1-\frac{\sqrt{\frac{1}{N} \Sigma _{i}^{N}(I_i-\bar{I})^2}/\bar{I}}{\sqrt{\frac{1}{N} \Sigma _{i}^{N}(I_{oi}-\bar{I_o})^2}/\bar{I_o}} \end{aligned}$$

where *I* is the pixel intensity, *N* is the number of pixel, $I_i$ is the local pixel intensity, $\bar{I}$ is the average pixel intensity, $I_o$ is the local pixel intensity at unmixed state, $I_{oi}$ is the local pixel intensity at unmixed state and $\bar{I_o}$ is the average pixel intensity at unmixed state. The MI is then plotted against the channel length and compared to the mixing efficiency of different patterns.

### Simulation

The simulation was performed with COMSOL Multiphysics. The simulated channel patterns were replicas of the actual devices. We used the laminar flow module. The size of meshing elements was chosen to be ranged from 0.461 to 7.07 $\upmu$m after we evaluated the convergence of data. We obtained the velocity and pressure profile for data analysis. The velocity profile helped us understand of movement of fluid elements with larger velocity while the pressure profile helped us understand how the radial pressure changed and perturbed along the horizontal mid-line. This work focused on experimental observation and simulation acted as an assistance to analyze the flow pattern we observed. The simulation was not identical to the reality in some conditions, for example, the fluid property was chosen the same as water, and the boundaries were considered the same. The $\Omega$ was plotted against the channel length with the simulated data. All the details of the simulation are attached in the supplementary document.

## Conclusion

The first deka-micron experimental study on Dean’s instability and mixing carried out on high aspect ratio channels is reported. Dean’s instability is achieved with a rolled-up velocity profile in the tortuous channel. Perturbation of smaller radii creates a different flow behaviour and makes the tortuous channel superior to the other channels regarding Dean’s instability creation at a lower *Re* and better mixing performance. Among the other three patterns, we conclude that the effect of the radius of curvature has more impact than the bending angle on mixing. The impact of Dean’s instability on particle sorting is predicted. This work demonstrates the promising ability for application in mixing and shows the potential for the utilization of Dean’s instability in particle sorting.

## Future prospect

This work shows the ability of tortuous channels to outperform the other channels in mixing. More investigation needs to be done for a more complete understanding of the dependency of flow rate on the mixing index. Different perturbations of radius in tortuous channels should also be tested if optimization is wanted. We showed a higher flow rate creates a more unstable environment and we predicted an increase in flow rate allows the complete mixing in a shorter channel length. The observed data also allows us to predict the effect of Dean’s instability on particle and cell separation. For normal Dean’s flow, particles are trapped by the two vortexes. We believe the rise of Dean’s instability has two possible impacts on particle focusing position. The first possibility is the shift of focusing position towards the pressure gradient-generated vortexes. The second possibility is the creation of extra focusing positions near the pressure gradient-generated vortexes as the De increases significantly.

### Supplementary Information

Supplementary Information.

## Data Availability

The data used in this study are available from the corresponding author upon reasonable request.
