# Supplementary material for: Flow study of Dean’s instability in high aspect ratio microchannels
Source: Sci Rep. 2023 Oct 19;13:17896. doi: 10.1038/s41598-023-44969-9 (PMC10587195; doi:10.1038/s41598-023-44969-9)
Supplement: Supplementary file 1 — Supplementary Information. [file 41598_2023_44969_MOESM1_ESM.pdf]

Supplmentary document

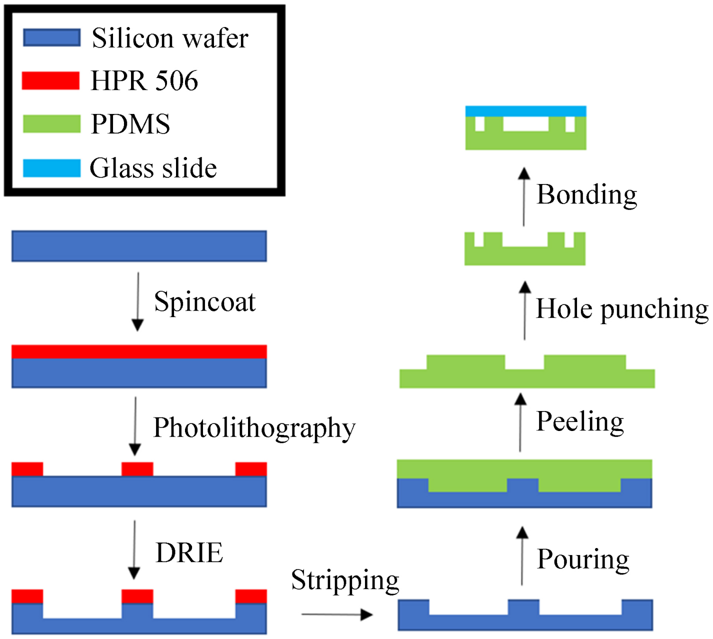

Figure 1. Fabrication process flow of the PDMS-based device

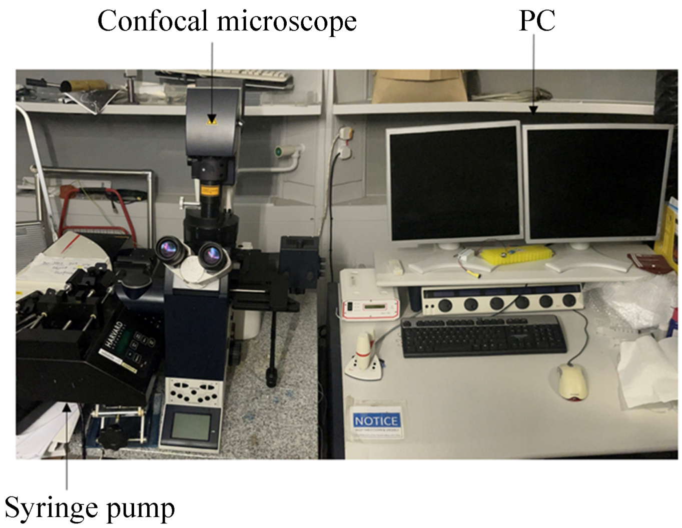

Figure 2. Experimental setup

Confocal microscopy parameters	
Software	LAS AF
Dimension	1024 times 512
Objective	40x
Laser	Argon
Laser power (%)	24
Excitation wavelength (nm)	458
Received wavelength (nm)	468 to 568
Gain (V)	900 to 1000
offset (%)	-0.2
Pinhole (µm)	80 to 125
Zoom	1.75x
Line accumulation	4
Line average	1
Frame accumulation	1
Frame average	4
Z-axis step size (µm)	1.83

Figure 3. Confocal microscopy parameters

Simulation parameters	
Physics model	laminar flow
Material	water
inlets flow rate (µl/min)	(500,500)µl/min and (1000,1000)µl/min
Wall condition	no slip
Meshing element sizes (µm)	0.461 to 7.07
Study	stationary, time dependent
Studied parameters	velocity, pressure

Figure 4. Simulation parameters
